# Dosimetric characteristics of dual‐layer multileaf collimation for small‐field and intensity‐modulated radiation therapy applications

**DOI:** 10.1120/jacmp.v9i2.2709

**Published:** 2008-03-31

**Authors:** Yaxi Liu, Chengyu Shi, Patricia Tynan, Niko Papanikolaou

**Affiliations:** ^1^ Cancer Therapy and Research Center, Medical Physics Department University of Texas Health Science Center, Radiation Oncology Department San Antonio Texas U.S.A.

**Keywords:** Multileaf collimator, dual‐layer MLC, Cerrobend block, penumbra width, planar dose difference, leaf‐end transmission

## Abstract

The purpose of the present work was to measure the performance characteristics in the penumbra region and on the leaf‐end of an innovative dual‐layer micro multileaf collimator (DmMLC). The DmMLC consists of two orthogonal (upper and lower) layers of leaves; a standard MLC consists of one layer. The DmMLC provides unique performance characteristics in smoothing dose undulation, reducing leaf‐end transmission, and reducing MLC field dependence of the leaf stepping angle. Two standard MLCs (80‐leaf and 120‐leaf versions: Varian Medical Systems, Palo Alto, CA), a DmMLC (AccuKnife: Initia Medical Technology, Canton, MA), and a Cerrobend (Cerro Metal Products, Bellefonte, PA) block were used in performance studies involving a triangular field, a cross leaf‐end field, and a circular field. Measurements were made with 6‐MV X‐rays and extended dose range film at a depth of 5 cm in Solid Water (Gammex rmi, Middleton, WI) at a source–axis distance of 100 cm. The field penumbra width measured between the 20% and 80% isodose lines through the MLC‐80, MLC‐120, DmMLC, and Cerrobend block were 9.0, 5.0, 3.0, and 2.0 mm respectively. The dose undulation amplitude of the 50% isodose line was measured as 5.5, 2.0, and 0.5 mm for the MLC‐80, MLC‐120, and DmMLC respectively. The planar dose difference between the MLC‐80, MLC‐120, and DmMLC against Cerrobend block was measured as ranging at ±52.5%,±35.0%, and ±20.0% respectively. The leaf‐end transmission was measured at 22.4% in maximum and 15.4% in average when closing a single layer of the DmMLC, and at 2.4% in maximum and 2.1% in average when closing both layers. The MLC dependence of the leaf stepping angle with the DmMLC ranged from 45 degrees to 90 degrees. The standard MLC leaf stepping angle ranged from 0 degrees to 90 degrees. In conclusion, the dose undulation, leaf‐end transmission, and MLC field dependence of the leaf stepping angle with the DmMLC were remarkably reduced as compared with those of the standard MLCs. And as compared with Cerrobend block, the DmMLC provided very comparable performance in field‐edge smoothing and in the shaping of complex fields.

PACS numbers: 87.56.Jk, 87.56.Nk, 87.56.Nj, 87.57.Nt

## I. INTRODUCTION

Multileaf collimators (MLCs) are used in place of Cerrobend (Cerro Metal Products, Bellefonte, PA) blocks to provide an efficient way of shaping treatment fields in radiotherapy.^(^ ^1^ ^–^ ^13^ ^)^ In conventional radiotherapy, MLCs can typically provide penumbra widths on the order of 0.5 cm to 1 cm at isocenter. That width may be too coarse for small fields such as those used for brain tumors or boost fields in the head and neck.

Several miniature or micro MLCs (mMLCs) have been developed and studied as a means of providing finer resolution for field margins.^(10,14–33)^ These mMLCs may provide an alternative strategy for reducing the dose to normal tissue in stereotactic radiosurgery^(15,20,27,29,31–33)^ and stereotactic radiotherapy,^(^ ^19^ ^,^ ^26^ ^,^ ^28^ ^)^ but although the thin leaf width of mMLCs improves field shaping, it still produces jagged field shapes because of finite leaf width. For intensity‐modulated radiation therapy (IMRT), the standard single‐layer MLC has leaf‐end transmission of 20%−30%.^(^ ^34^ ^,^ ^35^ ^)^ This leakage through the leaf ends could become significant in IMRT,^(35)^ resulting in “hot spots” between neighboring beam segments during step‐and‐shoot IMRT dose delivery.^(34)^

To reduce the stepped field‐edge effect when using a MLC, various solutions^(^ ^6^ ^,^ ^36^ ^)^ have been proposed. Galvin et al.^(6)^ proposed using a MLC and an isocenter shift‐smoothing method for superimposing fields. The proposed method implemented a combination of couch and leaf shifts to smooth the stepped dose distribution at the field edge and to improve the smoothness of the isodose lines. Woo and Nico^(37)^ studied the isocenter shift method on a Siemens HD270 (Siemens Medical Solutions, Malvern, PA) and demonstrated a clinical application in reducing dose undulation. As Galvin and colleagues^(6)^ pointed out, the magnitude of the reduction in the effective penumbra was small, and couch and leaf shifts can cause low‐dose regions to infringe on the treatment volume or a high dose to be given to critical structures on the field edge.

Evans and Partridge^(36)^ proposed rotating the collimator by 90 degrees to obtain two orthogonal beams. Their method entailed decomposing the required high‐resolution fluence distribution into two orthogonal components. Delivery was conducted using two leaf sweeps, with a collimator rotation of 90 degrees between the sweeps. That approach reduced the dosimetric effects at the field edge; however, significant overdosage^(36)^ at the edge of the field and dose leakage through the leaves was found to limit the usefulness of the method.

To reduce the stepped edge effect and the leaf‐end transmission, a novel dual‐layer micro MLC was developed. This device improves the accuracy of field shaping and reduces leaf‐end transmission by providing orthogonal upper‐ and lower‐layer multileaf collimation. The travel of the upper and lower layers of dual‐layer MLC leaves is orthogonal layer‐to‐layer and perpendicular to the central axis. This new device requires neither collimator rotation nor couch shifts to smooth the isodose lines at the field edge.

## II. METHOD AND MATERIALS

### A. DmMLC

The AccuKnife DmMLC by Initia Medical Technology (Canton, MA) was the first dual‐layer MLC of its kind to be approved for clinical use by the U.S. Food and Drug Administration. For the present study, the AccuKnife was mounted on a Varian 600C linear accelerator that has no built‐in MLC. The distance from the DmMLC platen to isocenter was 42.4 cm. For comparison, two standard MLC machines (a Varian Clinac 23EX with an 80‐leaf MLC and a Varian Clinac 2100C/D with a 120‐leaf Millennium MLC, hereafter called MLC‐80 and MLC‐120 respectively) were used to form the same fields to study the effect of leaf edge in the penumbra region. All MLC fields were compared with fields shaped using Cerrobend blocks.

**Figure 1:**
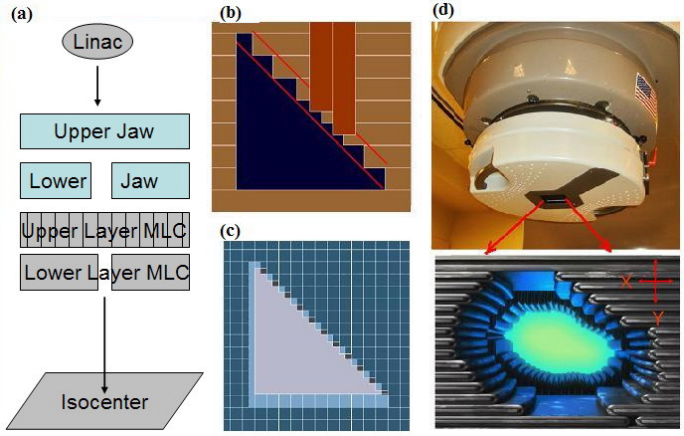
Illustrations of the dual‐layer multileaf collimator (MLC). (a) Schematic. (b) Layout of a lower‐ and an upper‐layer leaf. (c) Beam‐eye view of dual‐layer micro‐MLC (DmMLC) shaping a triangular field. (d) DmMLC mounted on a Varian Clinic 600C and axial view of dual‐layer leaves.

Fig. 1(a) is a simplified schematic of the DmMLC leaf layout. Fig. 1(b),(c) shows how the dual‐layer MLC forms a right triangular field. Fig. 1(d) shows the experimental setup, with the DmMLC mounted on the Varian 600C. The upper‐layer leaves (far from the patient) travel along the y‐axis and the lower‐layer leaves (close to the patient) travel along the x‐axis. The DmMLC studied in the present work has 96 tungsten leaves divided into four banks, each consisting of 14 inner leaves and 10 outer leaves with leaf widths at isocenter of 3.2 mm (lower layer) and 3.6 mm (upper layer) for the 14 inner pairs and 5.5 mm (lower layer) and 6.2 mm (upper layer) for the 10 outer pairs of lower and upper leaves. Each leaf has a height of 37.5 mm and a length of 60 mm. The maximum possible field size is 97×108 mm. The thin leaf width at isocenter and the orthogonal design of the upper and lower layers provide a capability for precision aperture shaping and complex field shaping.

### B. Evaluation patterns

Three evaluation patterns (right triangular, cross, and circular) were studied to demonstrate one performance characteristic of the DmMLC as compared with the standard single‐layer MLCs. The right triangular pattern field formed by the DmMLC was compared with nearly identical fields formed by the conventional MLCs and by Cerrobend blocks. The cross pattern field (straight leaf‐end and cross leaf‐end) was generated individually using the single layers and jointly using both layers of the DmMLC. The circular pattern field formed using both layers of the DmMLC was compared with the same field formed using only a single layer of the DmMLC.

**Figure 2:**
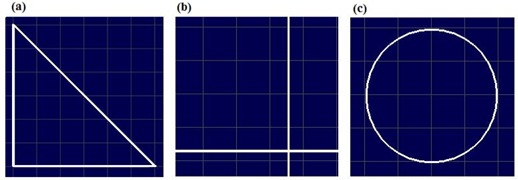
Evaluation shaping fields for multileaf collimator (MLC) and dual‐layer micro‐MLC (DmMLC). (a) A right‐triangular field formed by MLC, DmMLC, and Cerrobend (Cerro Metal Products, Bellefonte, PA) blocks. (b) A cross leaf‐end pattern for DmMLC. (c) A circular field.

The first field, a 45×45×90‐degree right triangular field [Fig. 2(a)] was used to investigate dose undulation at the field edge with the DmMLC. Theoretically, the effect of undulation at the field edge can be reduced by using two conventional MLC fields, one rotated 90 degrees from the other. This collimator rotation method has some disadvantages, including the possibility of significant overdosage at the edge of the field and of radiation leakage thought the leaves.^(34)^ It also requires highly accurate collimator rotation, with a change in the position of the indicated center of no more than 1 mm. With the use of DmMLC, the same goal can be achieved without collimator rotation.

The cross pattern was used to investigate leaf‐end transmission for a single‐layer and a dual‐layer closed leaf through the same device. The cross pattern actually consisted of two individual subpatterns—namely, a single‐layer closed pattern and a dual‐layer closed pattern. In the single‐layer closed pattern, a straight line of leaf‐end was formed by closing only one layer of DmMLC and keeping the other layer open, in a pattern similar to that of the standard MLC. In the dual‐layer closed pattern, a cross leaf‐end was formed by closing the upper‐layer and lower‐layer leaves as shown in Fig. 2(b).

The circular pattern was used to investigate the MLC dependence of the leaf stepping angle with the DmMLC. The circular field was 4 cm in diameter, as shown in Fig. 2(c). The field was first shaped using a single layer of the DmMLC to emulate the field shaping of a single‐layer MLC. The field was then reshaped using both layers.

Additionally, to demonstrate the complex field‐shaping capacity of dual‐layer MLC, a single segmental DmMLC field was used to generate a complex field of five adjacent circles. It may not be feasible to generate that kind of complex field with the use of any conventional single‐layer MLC.

### C. Penumbra and leaf‐end transmission measurements

Kodak extended dose range films (EDR2: Eastman Kodak Company, Rochester, NY) were used to measure the beam penumbra and planar dose. The film was positioned at the isocenter (source–axis distance: 100 cm) with 5 cm of Solid Water (Gammex rmi, Middleton, WI) phantom for buildup and 20 cm of Solid Water phantom as backscatter. All the radiographic films of various field shapes were irradiated using 6‐MV X‐rays. The films were developed using a Kodak X‐OMAT 5000 RA processor (Eastman Kodak Company) and scanned using a VXR‐16 Dosimetry Pro film scanner (Vidar Systems Corporation, Herndon, VA). A film calibration curve was created using the MLC step calibration method described in the RIT user guide, and RIT113 v. 4.4 (Radiological Imaging Technology, Colorado Springs, CO) was used to perform absolute dosimetry with the films. Isodose lines were plotted, and the effective penumbra of each MLC‐shaped field was measured as the distance between the peak of the 80% isodose line and the corresponding valley of the 20% isodose line. The dose undulation amplitude on the 50% isodose line was also measured. The leaf‐end transmission of the single‐layer leaf end and the cross leaf end were measured. The maximum and average leaf‐end transmissions for the cross pattern were measured and compared.

### D. Planar dose subtraction

Planar dose subtraction was used to compare differences in the dose distributions of image pairs. The difference plot is based on pixel‐by‐pixel subtraction of pairs of co‐registered images. This type of plot is capable not only of showing the amplitude of the dose difference between two images, but also of displaying areas where differences between the images exist.

When two images were registered, a cross‐boundary placement was applied. Cross‐boundary placement is a widely used method of conforming MLC leaves to a contour. The exact MLC leaf positions used were based on a variety of criteria. Typically, three methods of leaf placement are possible: in‐boundary placement, out‐boundary placement, and cross‐boundary placement.^(^ ^5^ ^,^ ^38^ ^)^ We used cross‐boundary placement in this study when comparing images between the MLCs, the DmMLC, and Cerrobend blocks.

## III. RESULTS

### A. Penumbra measurements and dose undulation amplitude

**Figure 3:**
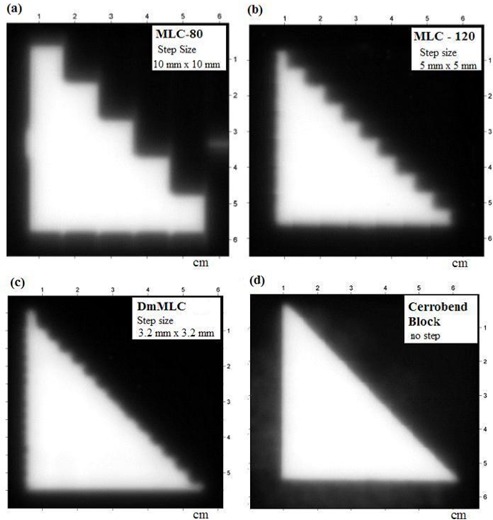
Radiographic films irradiated with 6 MV X‐rays. (a) Multileaf collimator (MLC)–80 with 1‐cm leaf width. (b) MLC‐120 with 0.5‐cm leaf width. (c) Dual‐layer micro‐MLC with 0.32‐cm leaf width. (d) Cerrobend (Cerro Metal Products, Bellefonte, PA) blocks.

Fig. 3(a,b,c,d) shows the right triangular fields separately shaped by MLC‐80, MLC‐120, DmMLC, and Cerrobend blocks as captured on radiographic films irradiated with 6‐MV X‐rays. Fig. 3 clearly indicates that the DmMLC approximated the triangular field much more closely than did the other MLC types.

**Figure 4:**
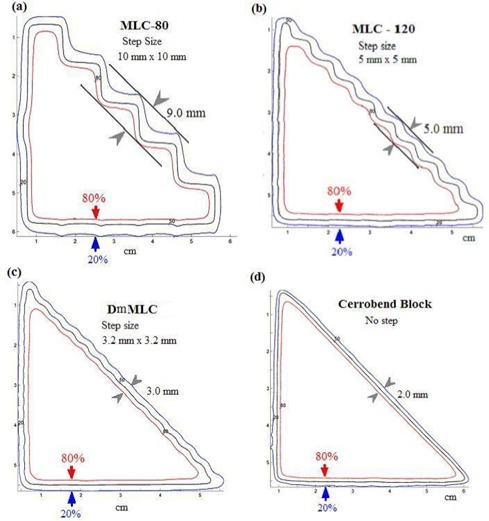
Penumbra measurements of 20%−80% isodose lines, and the dose undulation amplitude of 50% isodose line, (a) Multileaf collimator (MLC)–80 with 1‐cm leaf width. (b) MLC‐120 with 0.5‐cm leaf width. (c) Dual‐layer micro‐MLC with 0.32‐cm leaf width. (d) Cerrobend (Cerro Metal Products, Bellefonte, PA) blocks.

Fig. 4(a,b,c,d) presents the isodose lines from the triangular test pattern shaped by, respectively, the MLC‐80, MLC‐120, DmMLC, and Cerrobend blocks. The measured penumbra width between the 20% and 80% isodose lines was 9.0 mm for the MLC‐80, 5.0 mm for the MLC‐120, 3.0 mm for the DmMLC, and 2.0 mm for the Cerrobend blocks. The dose undulation amplitude of the 50% isodose line was measured as 5.5, 2.0, and 0.5 mm for the MLC‐80, MLC‐120, and DmMLC respectively. Table 1 summarizes the penumbra and dose undulation amplitude measurements.

Because of beam divergence and the fact that the two layers of the DmMLC banks are located at different distances from the source, the dosimetric characteristics of the beam are slightly different for the upper and lower layers of the DmMLC. Fig. 3(c) shows slightly less dose undulation at the middle of the 45‐degree field edge than is seen in Fig. 4(c).

**Table 1:** Penumbra, amplitude, and planar dose difference of the multileaf collimator (MLC)‐80, MLC‐120, dual‐layer micro‐MLC (DmMLC) and Cerrobend^a^ blocks

		*80%–20% Isodose line penumbra*	*50% Isodose line amplitude*	*Planar dose difference range at field edge* (±2.5%)	
*Collimation device*	*Leaf width (mm) at isocenter lever*	*[mm* (±0.25 mm)] *at 45‐degree angle*	*[mm* (±0.25 mm)] *at 45‐degree angle*	*against blocks*	*against DmMLC*
MLC‐80	10	9.0	5.5	52.5%	52.5%
MLC‐120	5	5.0	2.0	35.0%	35.5%
DmMLC	3.2	3.0	0.5	20.0%	—
Cerrobend block	—	2.0	0	—	—

a Cerro Metal Products, Bellefonte, PA.

### B. Leaf‐end transmission

The dual‐layer MLC design, with its overlapping orthogonal upper‐layer and lower‐layer MLC leaves, can reduce both interleaf and intraleaf transmission. Leaf‐end transmission was significantly reduced because one layer's leaf ends were always blocked by the other layer of leaves in the DmMLC.

**Figure 5:**
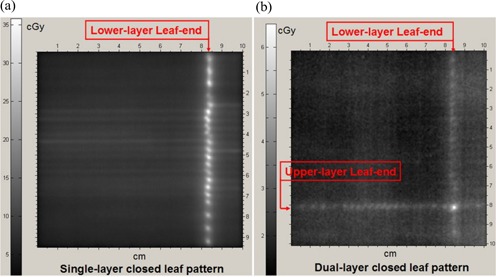
Radiographs for single‐layer and dual‐layer multileaf collimator (MLC) leaf‐end dose leakage with 6‐MV X‐rays. (a) Single‐layer closed pattern with a maximum leaf‐end transmission of 35.5 cGy. (b) Dual‐layer closed pattern with a maximum leaf‐end transmission of 6.5 cGy.

Radiographic films were irradiated with the same quantity of 6‐MV X‐rays for the single‐layer closed pattern and the dual‐layer closed pattern. (Fig. 5a,b) shows the films for the two test patterns. There is no special reason to choose the lower‐layer leaves for the single‐layer closed pattern, because the lower‐ and upper‐layer leaves are of identical design.

We first evaluated the range of leaf‐end transmission for the two test patterns. In the open field, we measured a 150‐cGy dose averaged over the film. The maximum leaf‐end transmission from the single‐layer closed pattern measured 35.5 cGy. The corresponding value for the dual‐layer closed pattern was 6.5 cGy. The measured interleaf transmission was much smaller than the intraleaf transmission in both test patterns. The interleaf transmission was measured to be less than 0.5% averaged over the central vertical profile from Fig. 5(b).

**Figure 6:**
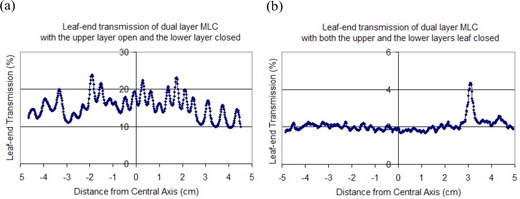
Leaf‐end transmission of multileaf collimator (MLC) and dual‐layer micro‐MLC. (a) Single‐layer closed pattern. (b) Dual‐layer closed pattern.

To further analyze dose leakage, we plotted the vertical dose profile of the leaf ends for the single‐layer closed pattern [Fig. 6(a)] and for the dual‐layer closed pattern with the leaf‐end transmission value normalized to 150 cGy [Fig. 6(b)]. For the single‐layer closed pattern, Fig. 6(a) shows a maximum leaf‐end transmission of 22.4% at 6 MV and an average value of 15.4%. For the dual‐layer closed pattern, Fig. 6(b) presents a maximum leaf‐end transmission of 2.4% at 6 MV, with an average value of 2.1%. In Fig. 6(b), the nominal maximum leaf‐end transmission is 4.3%. For the DmMLC, the cross point of the two layers of leaf ends—that is, Fig. 5(b)—is always inside the field. The effective maximum leaf‐end transmission is therefore the second maximum leaf‐end transmission in Fig. 6(b), which is 2.4%.

### C. MLC field dependence of leaf stepping angle

**Figure 7:**
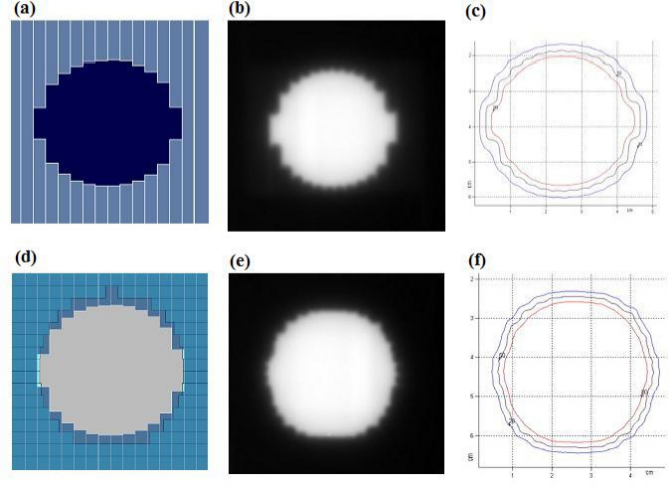
Circular field shaping by single‐layer and dual‐layer leaves of dual‐layer micro‐multileaf collimator (DmMLC). (a) Beam‐eye view (BEV) using single‐layer leaves of the DmMLC. (b) Radiograph by single‐layer leaves of DmMLC. (c) Isodose lines of circular field shaped by single‐layer leaves of DmMLC. (d) BEV of dual‐layer leaves of DmMLC. (e) Radiograph by dual‐layer leaves of DmMLC. (f) Isodose lines of circular field shaped by dual‐layer leaves of DmMLC.

The benefit of reducing the dependence of the leaf stepping angle can be shown with the circular field of 4 cm diameter (Fig. 7). Using a conventional MLC to shape the field, the MLC leaves are typically positioned as shown in Fig. 7(a). Fig. 7(b) shows a radiographic film of the circular field obtained using a single layer of the DmMLC. The effective penumbra was 2.5±0.25 mm and the stepping angle was 85.0±1.0 degrees on the top and bottom sides of the circle [Fig. 7(c)]. The effective penumbra on the left and right sides of the circle was 4.25±0.25 mm, and the stepping angle was 28.5±1.0 degrees.

Fig. 7(d),(e),(f) shows the beam's eye view of the MLC shape, the radiographic film image, and the isodose lines of the circular shape generated using both layers of the DmMLC. The effective penumbra at the top and bottom of the circle in Fig. 7(f) measured 2.5±0.25 mm, and the stepping angle measured 85.0±1.0 degrees. The effective penumbra on the left and the right sides of the circle measured 2.5±0.25 mm, and the stepping angle measured 85.0±1.0 degrees.

## IV. DISCUSSION

### A. DmMLC field‐edge smoothing

**Figure 8:**
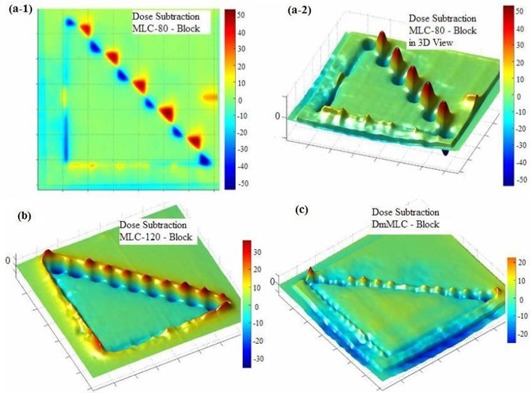
Planar dose comparison of multileaf collimator (MLC) subtracted by the Cerrobend (Cerro Metal Products, Bellefonte, PA) blocks. (a‐1) MLC‐80 against Cerrobend blocks in two‐dimensional view. (a‐2) MLC‐80 against Cerrobend blocks in three‐dimensional (3D) view. (b) MLC‐120 against Cerrobend blocks. (c) Dual‐layer micro‐MLF (DmMLC) against Cerrobend blocks.

Fig. 8 shows the results of planar dose subtraction for MLC‐80, MLC‐120, and DmMLC against Cerrobend blocks. The intensity at each point represents the magnitude of the difference between the dose at that point (MLCs) and the expected dose (Cerrobend blocks). The scale bar in Fig. 8 indicates the percentage of the normalized planar dose subtraction. (Fig. 8a‐1) indicates a dose difference of up to ±52.5% at the field edge for the MLC‐80 as compared with Cerrobend blocks. For a clearer view, we created a three‐dimensional (3D) plot of the planar dose difference. (Fig. 8a‐2) presents a 3D view of the planar dose difference plot between the MLC‐80 and Cerrobend blocks. Figs. 8(b) and 8(c) respectively present the planar dose differences plotted for the MLC‐120 and the DmMLC against Cerrobend blocks. Fig. 8(b) shows a dose difference of ±35% at the field edge for MLC‐120 against Cerrobend blocks. Fig. 8(c) shows a planar dose difference of ±20% at the field edge for DmMLC against Cerrobend blocks. Table 1 summarizes the planar dose subtraction results and the penumbra measurements.

**Figure 9:**
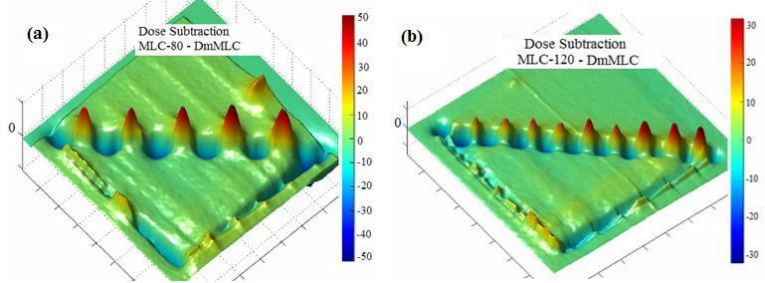
Planar dose comparison of multileaf collimator (MLC) subtracting dual‐layer micro‐MLF (DmMLC). (a) MLC‐80 against DmMLC. (b) MLC‐120 against DmMLC.

Additionally, for a direct comparison of MLCs, we plotted the planar dose difference between the MLC and DmMLC. Fig. 9(a) shows a planar dose difference ranging up to ±52.5% at the field edge for the MLC‐80 as compared with the DmMLC. Fig. 9(b) shows a planar dose difference ranging up to ±35.5% at the field edge for the MLC‐80 as compared with the DmMLC. Table 1 also summarizes the results of planar dose subtraction between the various MLCs.

### B. DmMLC reduces leaf‐end transmission

**Figure 10:**
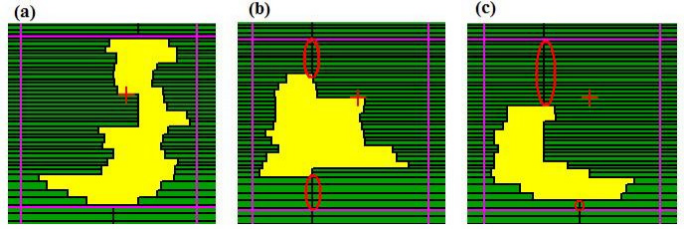
Multileaf collimator (MLC)–120 intensity‐modulated radiation therapy (IMRT), three segmentations in one field. (a) A segmentation of IMRT field matching the jaw settings. (b–c) Two additional segmentations in the same IMRT field.

For linear accelerator delivery of IMRT, multileaf collimation is used to conform the dose to the target volume, minimizing the amount of radiation absorbed by normal tissue. However, a standard single‐layer MLC has unavoidable leaf‐end transmission of 20% – 30%. The jaws were set to match the maximum field, as in Fig. 10(a). Fig. 10(b),(c) presents an additional two segments in the same IMRT field. In those panels, the radiation leakage area, where significant leaf‐end transmission exists, is marked out using ellipses. This design can lead to “hot spots” between neighboring beam segments during step‐and‐shoot IMRT dose delivery.^(^ ^34^ ^,^ ^35^ ^)^

**Figure 11:**
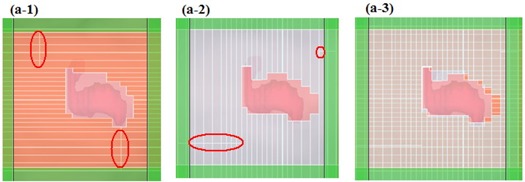
Dual‐layer micro‐multileaf collimator (DmMLC) intensity‐modulated radiation therapy (IMRT) in one segmentation. (a‐1) Beam‐eye view (BEV) of the lower‐layer MLC. (a‐2) BEV of the upper‐layer MLC. (a‐3) BEV of the DmMLC.

The dual‐layer MLC design turns out to be a solution well suited to minimizing leaf‐end transmission. Fig. 11 presents one segment of an IMRT field shaped by the DmMLC. (Fig. 11a‐1,a‐2) shows the lower‐layer MLC and the upper‐layer MLC for one IMRT field. Leaf‐end transmission occurred here as well and is marked with ellipses. When (Fig. 11a‐1) and (Fig. 11a‐2) are overlapped, the leaf‐end transmission is significantly reduced. (Fig. 11a‐3) demonstrates the single segmental DmMLC field.

### C. DmMLC reduces the MLC field dependence of the leaf stepping angle

**Figure 12:**
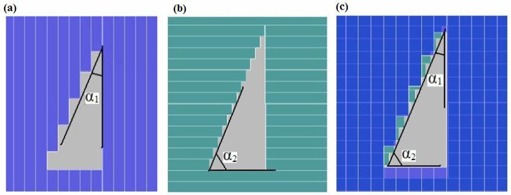
Schematic of multileaf collimator (MLC) fields with various stepping angles: (a) 27.5 degrees; (b) 62.5 degrees; (c) leaf‐stepping angle in dual‐layer micro‐MLC.

Various leaf stepping patterns can be used to shape MLC fields. The stepping angle is defined as the angle between the MLC leaf‐travel direction and the tangent line of the field edge curve. Considering the right triangle in Fig. 12, the leaf stepping angle is 27.5 degrees in Fig. 12(a), but 62.5 degrees in Fig. 12(b) when the collimator is rotated 90 degrees. It is not difficult to determine that Fig. 10(b) shows a finer approximation of the desired field than does Fig. 10(a). After studying the dependence of penumbra width on leaf stepping angle, Chow and colleagues^(^ ^39^ ^,^ ^40^ ^)^ concluded that penumbra widths are related to leaf stepping angles. It can be shown that the penumbra depends less on the stepping angle as that angle increases from 0 degrees to 90 degrees.

Because of the unique arrangement of dual‐layer orthogonal MLC leaves, the stepping angle definition needs some minor adjustments for the DmMLC. The stepping angle in the dual‐layer MLC is defined as the larger of the two stepping angles for the upper and lower leaves. For example, in Fig. 12(c), $\alpha_{1}$ is the stepping angle of the lower‐layer MLC (27.5 degrees) and $\alpha_{2}$ is the stepping angle of the upper layer (62.5 degrees). For the dual‐layer MLC, the worst scenario approaching the field edge occurs when the stepping angle is 45 degrees. The dependence of penumbra width on leaf stepping angle occurs only in the 45‐ to 90‐degree angle range.

### D. DmMLC enhances field‐shaping capability

Use of the DmMLC improves the effective penumbra and field shape conformity and reduces MLC transmission. Additionally, it allows for complex field shaping with the use of only one segment.

**Figure 13:**
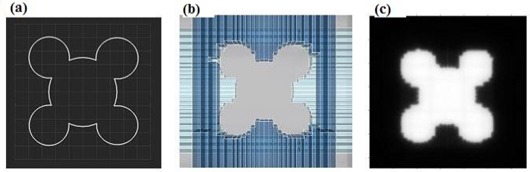
Complex field of five circles shaped by the dual‐layer micro‐multileaf collimator (DmMLC). (a) Drawing of the desired field shape. (b) Beam's eye‐view of dual‐layer leaves. (c) Extended dose‐range 2 film irradiated by the DmMLC.

The study by Evans and Partridge^(36)^ applied a circular field shape with single‐sweep and double‐sweep methods to analyze the dose distribution. In a double‐sweep field shape, the dose is delivered in two sets, with the two leaf sweeps differing by a 90‐degree collimator turn. Using the DmMLC, collimator rotation is not needed to obtain an equivalent circular field. As shown in Fig. 13(a,b,c), a single DmMLC field can be used to create even more sophisticated fields. With conventional MLCs, complicated field shapes are usually achieved in multiple segments. But the DmMLC can generate irregular field shapes in one segment by taking advantage of the dual‐layer mMLC configuration. The complex field shape used in this study consisted of a circle 5 cm in diameter, with four circles 2.5 cm in diameter arranged around the periphery. We selected that shape mainly to demonstrate the ability of the DmMLC to produce complicated field shapes.

Despite all the advanced features of the DmMLC discussed earlier, the DmMLC is not without its drawbacks. First, it is more difficult and expensive to manufacture than is a conventional single‐layer MLC. Second, the DmMLC has a limited field size of 10×10 cm, which means that it is suitable only for small tumors. Third, the DmMLC features of finer field shaping and extremely low leaf‐end transmission may not be of significant benefit to patients when a reduction in the dose to healthy tissue surrounding the tumor is not as critical as it is in head‐and‐neck cases.

## V. CONCLUSIONS

The results of the present study indicate that the DmMLC provides field shaping that is more precise at the field edges than is shaping by a standard MLC. The measurements of field penumbra width between the 20% and 80% isodose lines were 9.0 mm, 5.0 mm, 3.0 mm, and 2.0 mm for MLC‐80, MLC‐120, DmMLC, and Cerrobend blocks respectively. The dose undulation amplitude of the 50% isodose line was measured as 5.5 mm, 2.0 mm, and 0.5 mm for MLC‐80, MLC‐120, and DmMLC respectively. The planar dose difference range for MLC‐80, MLC‐120, and DmMLC against Cerrobend blocks was measured as ±52.5%, ±35.0%, and ±20.0% respectively. The maximum planar dose difference for MLC‐80 and MLC‐120 against DmMLC was measured as ±52.5% and ±35.5% respectively.

Leaf‐end transmission is significantly reduced with the DmMLC. The leaf‐end transmission for a single layer of the DmMLC was measured as a maximum of 22.4% and an average of 15.4% on the leaf end—values that were reduced to a maximum of 2.4% and an average of 2.1% when both layers of leaves were used. The interleaf transmission for the DmMLC was measured to be less than 0.5%.

Fields shaped by the DmMLC depend much less on the leaf stepping angle than do those shaped with a standard MLC. The leaf stepping angle for the DmMLC ranged from 45 degrees to 90 degrees; a standard MLC ranges from 0 degrees to 90 degrees. In addition, the DmMLC expands the capability for complex field shaping by offering orthogonal lower‐layer and upper‐layer MLC.

In summary, the DmMLC retains the general advantages of MLC while offering advantages in reducing field stepped edge effect, leaf‐end transmission, and MLC field dependence of the leaf stepping angle. It also enhances the possibility of using single segmentation for complex field shaping.

## ACKNOWLEDGMENTS

The work reported here was supported in part by a research grant from Initia Medical Technologies (Canton, MA). The authors acknowledge Martin Szegedi (University of Texas Health Science Center) for valuable discussion concerning this work.
